# Which elements were significant in reducing obstetric anal sphincter injury? A prospective follow-up study

**DOI:** 10.1186/s12884-021-04260-z

**Published:** 2021-11-18

**Authors:** Ole Bredahl Rasmussen, Annika Yding, Charlotte Sander Andersen, Jane Boris, Finn Friis Lauszus

**Affiliations:** Department of Obstetrics & Gynecology, Herning Hospital, Gl. Landevej 61, 7400 Herning, Denmark

**Keywords:** Obstetrics, Obstetrical anal sphincter injury, Perineum, Hands-on, Hands-off, Delivery

## Abstract

**Background:**

To examine which elements of an obstetric anal sphincter injury (OASI) care bundle were protective for OASI. Several interventional trials showed that application of a care bundle involving a hands-on approach to perineal protection may reduce the risk of OASI. Previously, we found that only the element “hand on the fetal head” in itself was protective, although the risk of a type 2 error was calculated to be 50%.

**Methods:**

A prospective follow-up study in an obstetric department in Denmark with 3200 deliveries per year. We included a cohort of 10,383 women giving birth vaginally from gestational week 22 + 0 from 2016 through 2019. We documented on a person-level the five elements of the care bundle together with maternal and obstetrical characteristics. The elements were 1) communication, 2) visible perineum, 3) hand on fetal head, 4) perineal support and 5) certification. Regression analysis was used for analysis of associations. The primary outcome measure was OASI.

**Results:**

The total rate of OASI in vaginally delivering women was 1.9%. The incidence was 3.2% in nulliparous women giving birth vaginally. The rate of cesarean section was 16.5% and for episiotomy 2.4%. The reduction in the incidence of OASI was sustained since 2013. Hand on the fetal head and perineal support both were protective factors for OASI. In case of a nulliparous woman with a neonate weighing 3500 g giving birth spontaneously, the relative risk (RR) for OASI was 0.50 (95% CI 0.49- 0.51) with use of hand on the fetal head together with perineal support against no use. Similarly, with a nulliparous woman giving birth to a neonate of 3500 g by vacuum extraction, the RR for OASI was 0.65 (95% CI 0.62-0.68) against no use.

**Conclusions:**

Both hand on the fetal head and perineal support were associated with a reduced risk of OASI.

## Background

Obstetric anal sphincter injury (OASI) is a serious complication of vaginal delivery. The severity of OASI is documented [1, 2] and confirmed by organizations representing women, such as “Mothers with Anal Sphincter Injuries in Childbirth” [3]. Suffering from an OASI is a strong risk factor for a negative birth experience [4].

Several interventional trials show that application of a care bundle involving a hands-on approach to perineal protection may reduce the risk of OASI [5–11]. Furthermore, several trials find that improved results with these interventions may be sustainable [12–14]. In one program, the specific use of manual perineal protection is documented and a protective effect found [15].

Previously, we showed that only the element of having a hand on the fetal head resulted in a statistically and clinically significant reduction in OASI [16], albeit the analysis as an exploratory pilot study was carried out on a modest-sized cohort; thus, the risk of a type 2 error was calculated to be 50%.

We aimed at analyzing the individual elements of an OASI care bundle including episiotomy and several known risk factors in a large cohort. Even though episiotomy may be viewed as a grade 2 laceration in itself, there is the potential that its use may reduce the risk of OASI [17]. Moreover, we wished to examine the sustainability over several years of the OASI care bundle. In general, a care bundle is meant to be implemented as a whole [18]. However, this may be complex in daily practice at a birth department since the ideal situation is not always present where all elements of the care bundle can be applied. Acute situations and specific birth positions and wishes by the woman giving birth may prevent the use of all elements. For this reason, we wished to analyze which separate elements of the care bundle were protective in order to provide evidence for a more thorough understanding of the value of each of the involved elements of the care bundle. In the broader perspective, there is a need for OASI research to focus more on delivery procedures and not restricting risk analyses to individual data on the woman or the infant [7].

## Methods

### Intervention

The present follow-up study arose from a quality improvement project that began in June 2013 at the delivery ward in Herning Hospital, Denmark in which we successfully reduced the incidence of OASI from 7.0 to 3.4% in vaginally delivering nulliparous women [9]. In this project, we implemented five elements of an OASI care bundle: 1) Communication settled; i.e. a shared decision with the woman, for instance about birth position and about not to push when the head is crowning; it is considered essential for complying with this element that the woman is explained about the intervention, understands the implications and confirms the use in the context of a shared decision making; the discussion and decision should preferably take place during pregnancy, but it may also take place during the first stage of labor; 2) Visible brim of the perineum during delivery of the fetal head in order to facilitate a view of perineum and the best possible application of hand on the fetal head and perineal support; 3) Whole hand on the fetal head as opposed to only using three fingers in order to slow down the speed of delivery when the head is crowning; 4) Perineal support during delivery of the head giving a firm support to perineum whilst simultaneously facilitating the naturally occurring extension of the head; 5) Certification of midwives, midwife-students and obstetricians to the OASI care bundle. The certification process is standardized using a power point presentation as a common starting point for introduction to the project and for further discussion. Furthermore, midwives practice the hands-on technique on a childbirth simulator and afterwards have three supervised births. Doctors practice operative vaginal deliveries on a childbirth simulator. Midwife supervisors are responsible for going through the introduction to the intervention and the certification process.

All women were included who gave birth vaginally in our department or at home with support by a midwife from our department from gestational age 22 + 0 in 2016 through 2019.

### Setting

In our ward we have approximately 3200 deliveries per year from gestational week 28 + 0. Very preterm births before this gestational age are transferred to a nearby university hospital at Skejby whenever feasible. Ninety full-time midwives and 20 doctors are working in our department. Information is given to the pregnant woman about what may be done to reduce the risk of OASI in consultations with the midwife in gestational week 29. A shared decision-making takes place about giving birth including preferred treatment of pain from contractions, birth position etc. In our department, instructions for midwives and doctors are that the naturally occurring extension of the head should be supported when the head is delivered through the introitus during spontaneous vaginal delivery (SVD) and operative vaginal delivery (OVD). In case of OVD, we use a vacuum extractor and we advocate that OVD is done as a team work with a midwife supporting the perineum while the doctor performs the extraction. Communication is maintained with the woman at all times. When the fetal head is crowning the doctor controls the speed of delivery using one hand on the extractor while the other regulates the necessary force and direction of the pull. Episiotomy is either by medio-lateral or by the lateral method. The latter is recommended in our department.

### Data collection

Information from each birth was entered into the electronic maternity records immediately after birth by the attending midwife. This included the use of each of the five elements of the care bundle. There was no registration of the time that each of the elements were applied. Satisfaction with the birth experience was recorded from September 2016 on a visual analogue scale from 1 to 10 where 10 is the highest level of satisfaction. The registration was carried out within the first couple of days after delivery. From December 2017 we started documenting birth position.

### Outcomes

The primary outcome was OASI, which comprises grade 3 and 4 anal sphincter injury according to the international classification used in our department for several years [19]. The attending midwife examines the perineum of the woman immediately postpartum including a digital rectal examination. Whenever the midwife suspects an OASI, a doctor is called to confirm the diagnosis. A midwife examines all women within 3 days postpartum to see how the tears are healing. In case of a missed OASI, a doctor changes the diagnosis accordingly. Women with an OASI receive follow-up care by a specialized midwife, physiotherapist and the gynecology clinic.

### Statistical analysis

Compared with the initial project we wanted to reduce the risk of a type-2 error and aimed at halving the variance. This meant that we needed at least four times the size of the population compared with the initial cohort of 1622 nulliparous women. Thus, all women giving birth vaginally from gestational week 22 + 0 from January 2016 through December 2019 were included in the cohort.

Statistics were performed with IBM SPSS Statistics 24. The difference between two means was tested with Student’s t-test if data followed Gaussian distribution; otherwise, Mann-Whitney’s test was used. χ2- test was used for categorical variables. Categorical variables were analyzed separately and by 5 elements present and absent combined. When the continuous variables age, gestational age, birth weight were categorized they were analyzed combined and for trend for linear association. Relative risk (RR) of OASI was calculated from a chosen baseline and stated with 95% confidence intervals (CI). Regression analysis was performed with OASI as dependent variable. Linear regression was used on the continuous variables age and birth weight and logistic regression on parity, the five elements individually (communication, visible perineum, hand on the fetal head, perineal support, certification), all five elements present (all-or-none), epidural analgesia, oxytocin drip, episiotomy, and vacuum delivery as they are dichotomous. All independent variables were tested individually against OASI and those with significant results and all five elements separately were added to a full model. Separate analyses were performed on all five elements present versus not all five elements present at birth. For multiparous women previous cesarean was added to the full model with all five elements individually. A two-sided *p* < .05 was the level of significance. Values are given as mean ± SD if Gaussian distributed or otherwise stated. We have adhered to the STROBE guideline when reporting this study.

## Results

During the 4 years, 12,429 women gave birth to 12,548 infants. The number of cesarean sections (CS) was 2047 resulting in a total rate of 16.5% with 17.3% in nulliparous women and 15.9% in multiparous (*p* < 0.04). From the total group we received feedback on birth experience from 6528 women; thus, the response rate was 61%. Satisfaction with birth experience was rated between 8 and 10 by 98%. In the group of vaginally delivering women, the rate of Apgar score < 7/5 min was less than 1% and the rate of umbilical cord pH < 7.0 was less than 0.5%. In the group with vaginal birth, 30% did not receive all five elements of the OASI care bundle. For instance, in 11% of deliveries the attending midwife was not previously certified to the care bundle. Likewise, 17% of deliveries took place without perineal support, while communication, visible perineum and hand on the fetal head were used in 90% of vaginal deliveries or more (Table 1).

**Table 1:** Characteristics of vaginal deliveries

		Nulliparous *N* = 4384	Multiparous *N* = 5999	Total *N* = 10,383
Age at delivery (years) ^a^ Mean (SD)		27.8 (4.2)	31.3 (4.3)**	29.9 (4.6)
Gestational age (days) Mean (SD)		279 (14)	280 (12)	280 (13)
Obstetrical factors N (%)	Spontaneous onset of delivery	3105 (71)	4466 (74)**	7571 (73)
	Dystocia treated with Oxytocin	704 (16)	326 (5.4)**	1030 (9.9)
	Epidural ^a^	1352 (31)	701 (12)**	2053 (20)
	OVD	549 (13)	136 (2.3)**	685(6,6)
	Episiotomy	198 (4.5)	55 (0.9)**	253 (2.4)
OASI care bundle N (%)	Communication	4211 (96)	5327 (89)**	9538 (92)
	Perineum visible	4114 (94)	5315 (89)**	9429 (91)
	Hand on fetal head	4172 (95)	5698 (95)	9870 (95)
	Perineal support	3800 (87)	4803 (80)**	8603 (83)
	Certification	3922 (89)	5284 (88)*	9206 (89)
	All five elements	3263 (74)	3961 (66)**	7224 (70)
Birth position ^b^ N (%)	Recumbent	601 (27)	800 (26)	1401 (26)
	Semi-recumbent	1272 (56)	1468 (47)**	2740 (51)
	Sitting upright / birthing chair	11 (0.5)	17 (0.5)	28 (0.5)
	Squatting	6 (0.3)	9 (0.3)	15 (0.3)
	Standing	28 (1.2)	70 (2.2)	98 (1.8)
	All fours	47 (2.1)	96 (3.1)	143 (2.7)
	Side lying	157 (6.9)	366 (11.7)**	523 (9.7)
	Water birth	144 (6.4)	303 (9.7)**	447 (8.3)
Condition of the newborn baby N (%)	Apgar < 7 / 5 min	39 (0.89)	48 (0.80)	87 (0.84)
	pH < 7.0 ^c^	15 (0.35)	13 (0.23)	28 (0.28)
	pH 7.0 – 7.09 ^c^	174 (4.1)	128 (3.8)	302 (3.4)
	Missed pH	121 (2.8)	221 (3.7)*	342 (3.4)
Birth weight (grams) ^d^ Mean (SD)		3470 (516)	3654 (512)**	3576 (522)

*SD* standard deviation, *OVD* operative vaginal delivery; ^a^information on age was missing in 391 cases and on epidural in 300 cases; percentages are calculated with the reduced denominator; ^b^ registration of birth position started by December 11, 2017; ^c^percentage calculated from total minus missed pH; ^d^singleton with gestational week ≥22 + 0. Nulliparous women versus multiparous: *: *p* < 0.05; **: *p* < 0.01. Herning Hospital, Denmark, 2016-2019

The total OASI rate in vaginal delivery was less than 2%. The risk of OASI for nulliparous women was almost fourfold the risk of multiparous women (RR = 3.67 (2.6-5.0) and OR = 3.76 (2.7-5.2)). Women with OVD had a 7.2 times higher risk of OASI compared with SVD (95% CI = 5.4-9.6; *P* < 0.0001). Similarly, the subgroup of multiparous women with a previous CS had a rate of OASI of 4.9%, which was 9.8 times higher compared with multiparous women without previous CS (95% CI = 5.7-16.7; *P* < 0.0001). The OASI rate was increased by nulliparity, epidural analgesia, dystocia treated with oxytocin, episiotomy, OVD, and higher birth weight. Birth weight increased the risk of OASI most prominently in nulliparous women. Standing birth position in nulliparous women was complicated by more cases with OASI (Table 2). The use of the single elements of the intervention together with the rate of OASI is shown in Table 3. Only very few cases occurred with just one specific element present and at the same time none of all other elements (less than 10 cases for four elements and only 71 cases for “Certification present”). For this reason, we only show data where just one element at a time was missing. Reduced rates were seen with visible perineum, hand on the fetal head and perineal support in the whole group and in the nulliparous group (Table 3).

**Table 2:** Incidence of OASI in subgroups in vaginal deliveries

Vaginal deliveries		Nulliparous			RR (95% CI)	Multiparous			RR (95% CI)	Total			RR (95% CI)
		n	OASI	%		n	OASI	%		n	OASI	%	
Parity		4384	142	3.2		5999	53	0.9**		10,383	195	1.9	
Maternal age at birth a) (years)	– 19	49	0	0	–	2	0	0	–	51	0	0	–
	20 – 24	816	16	2	1.0	263	0	0	–	1079	16	1.5	1.0
	25 – 29	2056	68	3.3	1.69 (0.9-2.9)	1701	10	0.6	1.0	3757	78	2.1	RR 1.4 (0.8-2.4)
	30 – 34	1011	38	3.8*	1.92 (1.1-3.4)	2541	28	1.1	1.87 (0.9-3.8)	3552	66	1.9	RR 1.25 (0.7-2.2)
	35 – 39	237	7	3.0	1.51 (0.6-3.6)	1062	12	1.1	1.9 (0.8-4.2)	1299	19	1.5	RR 0.99 (0.5-1.9)
	40 –	49	0	0		205	1	0.5		254	1	0.4	–
Sectio antea	No	–	–	–		5465	27	0.5	1.0	–	–	–	
	Yes	–	–	–		534	26	4.9**	9.8 (5.7-16.7)	–	–	–	
Gestational age (days)	< 266	400	1	0.3	0	399	1	0.3	–	799	2	0.3	–
	266 – 272	504	11	2.2	1.0	615	4	0.7	1.0	1119	15	1.3	1.0
	273 – 279	912	29	3.2	1.46 (0.7-2.9)	1260	10	0.8	1.22 (0.4-3.9)	2172	39	1.8	RR 1.34 (0.7-2.4)
	280 – 286	1334	44	3.3	1.51 (0.8-2.9)	2154	22	1.0	1.57 (0.5-4.5)	3488	66	1.9	RR 1.41 (0.8-2.5)
	≥ 287	1234	57	4.6*	2.12 (1.1-4)	1571	16	1.0	1.57 (0.5-4.7)	2805	73	2.6*	RR 1.94 (1.1-3.4)
Dystocia	No	3680	115	3.1	1.0	5673	44	0.8	1.0	9353	159	1.7	1.0
	Yes	704	27	3.8	1.19 (0.8-1.7)	326	9	2.8*	3.19 (1.7-5.8)	1030	36	3.5**	RR 2.06 (1.4-2.9)
Epidural a)	No	2984	94	3.1	1.0	5046	38	0.8	1.0	8030	132	1.6	1.0
	Yes	1352	47	3.5	1.07 (0.8-1.4)	701	15	2.1**	2.35 (1.52-3.6)	2053	62	3.0**	RR 1.86 (1.4-2.5)
Mode of delivery	SVD	3835	89	2.3	1.0	5863	40	0.7	1.0	9698	129	1.3	1.0
	OVD	549	53	9.7**	3.19 (2.5-4)	136	13	9.6**	11.9 (7.2-19.6)	685	66	9.6**	RR 7.2 (5.4-9.6)
Episiotomy	No	4186	129	3.1	1.0	5945	52	0.9	1.0	10,130	181	1.8	1.0
	Yes	198	13	6.6**	2.1 (1.2-3.6)	55	1	1.8	2.08 (0.3-14.7)	253	14	5.5**	RR 3.06 (1.8-5.2)
Birth position a)	Recumbent	601	23	3.8		801	7	0.9		1401	30	2.1	
	Semi-recumbent	1272	41	3.2		1468	16	1.1		2740	57	2.1	
	Sitting upright/birth chair	11	0	0		17	0	0		28	0	0	
	Squatting	6	0	0		9	0	0		15	0	0	
	Standing	28	4	14.3*		70	0	0		98	4	4.1	
	All fours	47	2	4.3		96	1	1.0		143	3	2.1	
	Side lying	157	4	2.5		366	3	0.8		523	7	1.3	
	Water birth	144	5	3.5		303	1	0.3		447	6	1.3	
	Sub-total	2266	79	3.5		3130	28	0.9		5395	107	2.0	
Birth weight (grams)	– 2999	820	19	2.3	1.0	730	4	0.5	1.0	1550	23	1.5	1.0
	3000 – 3499	1489	25	1.7	0.73 (0.4-1.3)	1636	10	0.6	1.12 (0.4-3.5)	3125	35	1.1	0.75 (0.4-1.3)
	3500 – 3999	1481	66	4.5*	1.92 (1.2-3.2)	2211	17	0.8	1.54 (0.5-4.6)	3692	83	2.2	1.52 (0.96-2.4)
	4000 –	594	32	5.4**	2.33 (1.3-4.1)	1422	22	1.5*	2.82 (0.98-8.2)	2016	54	2.7*	1.81 (1.1-2.9)

*SVD* Spontaneous vaginal delivery, *OVD* Operative vaginal delivery; Maternal age: groups vs. age group 20 – 24 years. Sectio antea: yes vs. no. Gestational age: groups vs. 266 – 272 days. Dystocia: yes vs. no. Epidural: yes vs. no. Mode of delivery: OVD vs. SVD. Episiotomy: yes vs. no. Birth position: standing vs. semi-recumbent and recumbent. Birth weight: groups vs. < 3000 g. a): Information on age was missing in 391 cases, on epidural in 300 cases, and on birth position in 4988 cases. *: *p* < 0.05; **: *p* < 0.01; Herning Hospital, Denmark, 2016-2019

**Table 3:** Intervention - the single elements of the OASI care bundle and the incidence of OASI according to parity

	Whole care bundle present		Communication present		Visible perineum present		Hand on fetal head present		Perineal support present		Certification present	
	No	Yes	No	Yes	No	Yes	No	Yes	No	Yes	No	Yes
Total group (no.)	3159	7224	845	9538	954	9429	513	9870	1780	8603	1177	9206
OASI (no.)	72	123	12	183	21	174	28	167	47	148	14	181
%	2.3	1.7 *	1.4	1.9	2.2	1.8	5.5	1.7 **	2.6	1.7 *	1.2	2.0
RR	1.0	0.75	1.0	1.35	1.0	0.84	1.0	0.31	1.0	0.65	1.0	1.65
95% CI		0.6-0.99		0.8-2.4		0.5-1.3		0.2-0.5		0.5-0.9		0.96-2.8
Nulliparous (no.)	1121	3263	173	4211	270	4114	212	4172	584	3800	462	3922
OASI (no.)	59	83	9	133	15	127	23	119	38	104	11	131
%	5.3	2.5 **	5.2	3.2	5.6	3.1 **	10.8	2.9 **	6.5	2.7 **	2.4	3.3
RR	1.0	0.48	1.0	0.61	1.0	0.55	1.0	0.26	1.0	0.42	1.0	1.4
95% CI		0.4-0.7		0.3-1.2		0.3-0.9		0.2-0.4		0.3-0.6		0.8-2.6
Multiparous (no.)	2038	3961	672	5327	684	5315	301	5698	1196	4803	715	5284
OASI (no.)	13	40	3	50	6	47	5	48	9	44	3	50
%	0.6	1.0	0.4	0.9	0.9	0.9	1.7	0.8	0.8	0.9	0.4	0.9
RR	1.0	1.6	1.0	2.1	1.0	1.0	1.0	0.5	1.0	1.2	1.0	2.3
95% CI		0.9-3.0		0.8-6.7		0.4-2.3		0.2-1.3		0.6-2.5		0.7-7.2

Element not present: All combinations of the elements of the care bundle except the element in question. Element present: All combinations of the elements in the care bundle including the element in question. *RR* relative risk, *CI* confidence interval. Yes vs. No: * = *p* < 0.05. ** = *p* < 0.01. Herning Hospital, Denmark, 2016-2019

Some of the elements showed association on the OASI rate, which was counter-intuitive, although the numbers were small. We carried out a subgroup analysis of the element “Certification”, which in the univariate analysis showed a non-significant tendency towards a smaller risk of OASI in deliveries without certified midwife in both parity groups. The subgroup analysis showed that non-certified midwives took care of fewer women with a previous CS, as well as fewer with epidural and dystocia treated with oxytocin. Likewise, the birth weight was significantly smaller in the multipara group where the midwife taking care of the delivery was not certified (data not shown).

Presence of the whole care bundle (all five elements) was associated with a significant reduction in the OASI rate in the whole group and in nulliparous women. To investigate further the association of the whole care bundle, we compared the event “all five elements” being present and “not all five elements” present with the incidence of OASI. In the group of nulliparous, the incidence of OASI was 5.3% vs. 2.5% in favor of the presence of the whole care bundle. Similarly, in the case of OVD, the OASI was 15.6% compared to 7.7% in favor of all five elements present (Table 4).

**Table 4:** Intervention – use of the care bundle in different categories and the incidence of OASI

		All 5 elements of care bundle present Total / OASI No. / no. (%)	Not all 5 elements of care bundle present Total / OASI No./ no. (%)	*p*-value
Parity	Nulliparous	3263 / 83 (2.5)	1121 / 59 (5.3)	Nulliparous: 0.001 ** Multiparous: 0.15 Combined: 0.046 *
	Multiparous	3961 / 40 (1)	2038 / 13 (0.6)	
Maternal age at birth a) (years)	– 19	41 / 0	10 / 0	Combined: 0.009** Linear trend: 0.009**
	20 – 24	787 / 10 (1.3)	292 / 6 (2.1)	
	25 – 29	2671 / 46 (1.7)	1086 / 32 (2.9)	
	30 – 34	2425 / 41 (1.7)	1127 /25 (2.2)	
	35 – 39	883 / 12 (1.4)	416 / 7 (1.7)	
	40 –	184 / 1	70 / 0	
Sectio antea	No	3549 /21 (0.6)	1916 /6 (0.3)	No: 0.16 Yes: 0.61 Combined: 0.15
	Yes	412 / 19 (4.6)	122 / 7 (5.7)	
Gestational age (days)	< 266	570 / 1 (0.2)	229 / 1 (0.4)	Combined: 0.046* Linear Trend: 0.046*
	266 – 272	788 / 10 (1.3)	331 / 5 (1.5)	
	273 – 279	1498 / 23 (1.5)	674 / 16 (2.4)	
	280 – 286	2370 / 41 (1.7)	1118 / 25 (2.2)	
	≥ 287	1998 / 48 (2.4)	807 / 25 (3.1)	
Dystocia treated with Oxytocin	No	6408 / 97 (1.5)	2945 / 62 (2.1)	No: 0.04 * Yes: 0.29 Combined: 0.046*
	Yes	816 / 26 (3.2)	214 / 10 (4.7)	
Epidural a)	No	5475 / 82 (1.5)	2555 / 50 (2)	No: 0.001 ** Yes: 0.1 Combined: 0.001 **
	Yes	1659 / 41 (2.5)	394 / 21 (5.3)	
Mode of delivery	SVD	6706 / 83 (1.2)	2992 / 46 (1.5)	SVD: 0.13 OVD: 0.003 ** Combined: 0.046*
	OVD	518 / 40 (7.7)	167 / 26 (15.6)	
Episiotomy	No	7044 / 114 (1.6)	3086 / 67 (2.2)	No: 0.053 Yes: 0.56 Combined: 0.046*
	Yes	180 / 9 (5)	73 / 5 (6.8)	
Birth weight (grams)	– 2999	1061 / 18 (1.7)	489 / 5 (1)	Combined: 0.046* Linear trend: 0.046*
	3000 – 3499	2178 / 18 (0.8)	947 / 17 (1.8)	
	3500 – 3999	2539 / 54 (2.1)	1153 / 29 (2.5)	
	4000 –	1446 / 33 (2.3)	570 / 21 (3.7)	

Categorical variables analyzed separately and combined. Continuous variables analyzed combined and with linear trend. a) Information on age was missing in 391 cases, on epidural in 300 cases, and on birth position in 4988 cases; *: *p* < 0.05; **: *p* < 0.01. *SVD* spontaneous vaginal delivery, *OVD* operative vaginal delivery. Herning Hospital, Denmark, 2016-2019

In the univariate regression analysis nulliparity, higher birth weight, epidural, dystocia treated with oxytocin, episiotomy, and OVD were associated with a higher risk of OASI. In contrast, hand on the fetal head, perineal support and the OASI care bundle with all five elements present were associated with a lower risk (Table 5).

**Table 5:** Selected variables with univariate and full model regression analysis, odds ratio and adjusted odds ratio of perineal tear grade 3 and 4 (OASI). Data for the whole group of women except when otherwise specified

	Univariate analysis				Full model analysis			
Variable	Coefficient (B)	*p*-value	Crude OR	95% CI	Coefficient (B)	*p*-value	Adjusted OR	95% CI
Age at delivery^a^	−0.009	0.4			–	–		
Parity (higher)	−1.3	0.001	0.27**	0.2-0.4	−0.94	0.0001	0.39**	0.3-0.6
Dystocia treated with oxytocin	0.7	0.001	2.09**	1.5-3	− 0.07	0.7	1.07	0.8-1.5
Epidural	0.6	0.001	1.86**	1.4-2.5	0.11	0.42	1.12	0.8-1.5
Episiotomy	1.2	0.001	3.22**	1.8-5.6	0.16	0.62	1.18	0.7-2.1
OVD	2.1	0.001	7.9**	5.8-10.8	1.7	0.0001	5.3**	3.9-7.2
Communication	0.3	0.31	1.24	0.8-2.4	0.34	0.83	1.41	0.9-2.7
Visible perineum	−0.18	0.44	0.84	0.5-1.3	−0.06	0.862	0.95	0.6-1.5
Hand on fetal head	−1.2	0.001	0.3**	0.2-0.5	−0.78	0.001	0.46**	0.3-0.8
Perineal support	−0.4	0.01	0.65*	0.5-0.9	−0.78	0.0001	0.47**	0.4-0.7
Certification	0.51	0.07	1.67	0.97-2.9	0.07	0.2	1.47	0.9-2.6
All five elements ^b^	−0.3	0.047	0.74*	0.6-0.99	−0.49	0.001	0.61**	0.5-0.8
Birth weight ^a^	0.061	0.0001			–	–		
*Multiparous only:*								
Previous cesarean	2.3	0.001	10.3**	6-17.8	1.9	0.001	6.8**	4-12

*OVD* operative vaginal delivery. ^a^ age and birth weight by linear regression (Beta coefficient) and not included in the full model; all other logistic regression (Coefficient (B); ^b^ All five elements present replacing the individual elements in the full model (i.e., communication, visible perineum, hand on head, perineal support and certification), *: *p* < 0.05; **: *p* < 0.01. Herning Hospital, Denmark, 2016-2019

For evaluation in the full regression model, all significant variables were entered from the univariate regression analysis and the five individual elements of the care bundle. Thus, dystocia treated with oxytocin and epidural were no longer significant factors. Nulliparity and OVD remained as factors increasing the risk of OASI. Still, hand on the fetal head and perineal support were associated with a reduction of the OASI rate, when adjusted for all other factors except age and birth weight.

From the full regression model, we could calculate the potential benefit of using the care bundle. For instance, in a case of a nulliparous woman with a neonate weighing 3500 g giving birth with SVD the RR for OASI is 0.50 (CI: 0.49; 0.51) with use of hand on the fetal head together with perineal support against no use. Similarly, with a nulliparous woman giving birth to a neonate of 3500 g with OVD the RR is 0.65 (CI: 0.62; 0.68) against no use.

## Discussion

### Main findings

The emphasis on documenting process indicators on a person-level enabled us to gain novel information on the quality of obstetrical care concerning the relation between the individual elements and the outcome. Both hand on the fetal head and perineal support were associated with less OASI as did the 5 elements of the care bundle together. This finding was in alignment with previous interventional programs using similar care bundles [5–11]. The comprehensiveness of the birth cohort together with the intention of applying the five elements to all women constitute a sound ground for concluding that the intervention was associated with a benefit for women giving birth.

The OASI care bundle has been used in our department since 2013. In the original project, we reduced the OASI rate to 3.4% in nulliparous women delivering vaginally. The incidence was 3.2% in the present period from 2016 through 2019. Thus, corresponding with previous studies on robustness [12–14] the reduction was sustained for more than 7 years and cannot be attributed merely to focus or a hypothetical Hawthorne effect.

Risk factors for suffering OASI in our study as well as in other’s were nulliparity, OVD and higher birth weight [20, 21]. These well-established risk factors were present before and after a comparable, successful interventional program [22]. They may be modified in strength by an OASI care bundle but most likely, not to the extent that the risks disappear.

The tendency towards less OASI when a non-certified midwife took care of the delivery (see Table 3) was surprising but in fact logical. In subgroup analysis, it turned out that the group of non-certified midwives were taking care of less complicated births, which is in accordance with our policy. Furthermore, most were at the same time supervised by a certified midwife. Some of the other subgroups with absent single elements of the care bundle contained too small numbers to justify firm conclusions due to type II error (Table 4).

In almost all variables the rate of OASI was less in women who had the whole care bundle applied (see Table 4). The difference in the OASI rate within the multiparous women was too small to warrant any safe conclusion. On the other hand, the absolute rate of OASI in multiparous in our study was comparable to other studies (7, 11, 13).

The use of episiotomy was associated with the risk of OASI (Table 1) but, mostly likely, not modified by the use of the bundle of care in this study (Table 4). Changing the concept is difficult about which clinical situations constitute an indication for episiotomy. In the recent EPITRIAL, one arm with routine care was compared with a study arm in which no episiotomy was to be performed [23]. The interim analysis surprisingly found that the rate of episiotomy was equal in the two trial arms. A probable explanation for this may be that several of the indications for episiotomy are subjective and based on tradition and previous experience by midwives and doctors. The most effective way to implement a desired change in habits is likely a systematic approach with shared procedures and continuous audit, where data on processes and outcomes are monitored and provided for the staff together with education on the background for the change [23, 24]. This type of a systematic, organizational approach is a key point in the quality improvement work in our department, as well [9]. A new UK trial, “OASI2”, will look more into scalable mechanisms of the implementation process of an OASI care bundle [25].

### Strengths and limitations

A strength of this prospective follow-up study is that the OASI care bundle was implemented 7 years ago and is an integrated part of the department. The attending midwife carries out documentation of the use of the elements of the OASI care bundle as soon as possible after the birth. Adherence to the care bundle is monitored and feedback is provided for the staff of the process and outcome indicators.

Standing birth position came out with a high OASI rate and, at the same time, constituted a challenge for the application of all five elements. In the present study, birth position in itself was not a significant factor for the risk of OASI after controlling for confounding factors. This is in alignment with other studies on risk factors for OASI [14, 26]. Choice of birth position only constitutes a potential limitation to the use of all elements of the care bundle during the last contractions before delivery. In fact, hand on the fetal head may be used in practically all birth positions. Informed choice of birth position lies with the delivering woman. Emphasis should be on supporting the woman as much as possible according to her preferences irrespective of the birth position.

The rate of asphyxia was similar compared with previous years and lower than the quality standards of ≤1.0%, set by the Danish Quality Database for Birth [27]. Likewise, the rate of episiotomy remained low. Restrictive use of episiotomy is endorsed by FIGO rather than its routine use [28]. It may be that the acceptable rate should be as low as 10%, although, the actual rate will, of course, reflect the baseline risk profile of the population. Besides imminent asphyxia, certain specific indications may stand the test in future research like episiotomy in OVD in nulliparous women [29], thereby, providing the ground for a common acceptance of the rate of episiotomy as an indicator of better quality of obstetrical care with optimum OASI rates [28].

The period where the elements of the intervention are applied (exposure time) should preferably be described in addition to presenting and analyzing the exposure and the outcomes in an interventional trial. In the present project, the time was not registered where the hands-on elements were in use because of the anticipated short period. This may constitute a weakness of the study. We advocate that the elements should only be applied during the last couple of contractions in order to avoid undue delay of the birth. In a case with suspected asphyxia, an expedite delivery will, of course, have first priority. In the beginning of the project, we noticed that hand on the fetal head and perineal support were applied for a longer period compared with later on in the project. This may still be an issue for untrained staff members. A too long application time of the hands may cause tiring of the hands besides possibly leading to an unwanted delay of the birth. For these reasons, we included this as a learning point for untrained midwives and doctors. A possible strength of these observations is an increased attention on being especially alert when the head is descending through the birth canal in order to avoid a sudden, uncontrolled fast delivery.

Another weakness in this study was the potential risk of residual confounding that was not taken into account by the statistical analysis. Causal relationships should be interpreted with caution because of the observational design, although, previously, we argued that the Bradford-Hill criteria support a causal relation between the OASI care bundle and a reduced risk of OASI [16]. The study was carried out in a single department and, therefore, external validity may be difficult to estimate. This may partly depend on the a priori attitude towards implementation of quality improvement work and the hands-on approach in a department.

### Interpretation

We acknowledge that all the elements of OASI care bundles should be seen as integrated parts of the combined bundles [11, 18]. In our case, five elements constitute the OASI care bundle. The first element concerning communication with the woman together with the fifth element concerning certification may be viewed as fundamental parts of a treaty between the mother and the midwife and/or the doctor. The second element of the care bundle concerning visible perineum is conditional for applying hands-on in the best possible way. Thus, the three elements may be perceived as facilitating the hands-on approach, where hand on the fetal head and perineal support constitute the active elements of the care bundle. This concept was supported by the findings in the present study and may add to the understanding of the effect of the care bundle and why it should be seen as a whole. Alternatively, as many elements should be applied as possible.

In the present study only around 70% of the women received all five elements of the OASI care bundle while 83% received perineal support (Table 1). Some women gave birth very quickly preventing the use of all elements. Some women preferred delivering in a position where not all elements could be applied. In a study by De Meutter only 73% of all deliveries received manual perineal protection [15]. This was partly ascribed to difficulties for specialists during OVD who applied perineal protection in less than half of the deliveries they attended. The explanation offered for this was emergencies with an urge to deliver the baby as fast as possible. Another suggestion was that professionals with more experience might have more difficulty to adapt to a new technique. Clinicians’ perspectives on the use of an OASI care bundle were analyzed in a recent study, which found that adoption of the OASI care bundle relied on several components [30]. Among these were the initial implementation strategy in a department, personal values concerning the possible need for changing behavior by the clinician and different perceptions of what women want. Since it is not feasible to apply all elements of an OASI care bundle in all deliveries, we find it important to document the use of each element, especially when analysis is carried out for comparative reasons.

In fact, the lack of analyzing per protocol may partly explain that no difference was found in the risk of OASI in the RCTs between the hands-on versus hands-off techniques. Three recent meta-analyses on randomized trials [31–33] include five RCT’s with data on OASI incidence [34–38]. When the intention-to-treat analyses in these trials are combined with defining the intervention in the hands-off arm as “hands-poised”, the treatment that was actually provided in each case becomes obscured, insofar the ‘hands-poised’ method allows for using elements from the hands-on method. It may be that treatments in both randomization arms in the RCTs were in reality quite similar, thus, explaining that no difference in outcome was found. We propose that the elements of the interventions that are used should be documented on a person-level, also in RCT’s.

Another issue in the discussion between hands-off versus hands-on is about the definition of the hands-on intervention. In the three larger RCT’s the supposed aim in the hands-on technique was to “increase flexion of the head” [34, 35] or “maintaining the flexion of the head” [38]. We are aware that the fetal head is flexed on its way through the birth canal. However, the head extends through the introitus when the head is crowning in the final part of the delivery. The distinction between flexing and extending the head is crucial and should be made clear in future work and meta-analyses in this field.

## Conclusions

The present study confirms that a lesser risk of OASI is associated with use of both hand on the fetal head and perineum. We suggest documenting on a person-level the specific elements of the intervention aimed at reducing the incidence of OASI. Future studies should focus on the question of flexion or extension of the head of the child during the passage of the introitus in order to achieve a further reduction in OASI.

## Data Availability

The anonymized data that support the findings of this study are available from the corresponding author upon reasonable request.

## Abbreviations

OASI: Obstetric anal sphincter injury
CS: Caesarean section
SVD: Spontaneous vaginal delivery
OVD: Operative vaginal delivery
RR: Relative risk
CI: Confidence interval
